# Nonlinear Control Strategies for an Autonomous Wing-In-Ground-Effect Vehicle

**DOI:** 10.3390/s21124193

**Published:** 2021-06-18

**Authors:** Davide Patria, Claudio Rossi, Ramon A. Suarez Fernandez, Sergio Dominguez

**Affiliations:** 1 Politecnico Di Torino, 10129 Torino, Italy; davide.patria@gmail.com; 2 Centre for Automation and Robotics UPM-CSIC, Universidad Politécnica de Madrid, 28006 Madrid, Spain; claudio.rossi@upm.es (C.R.); ramon.suarez@upm.es (R.A.S.F.)

**Keywords:** Unmanned Surface Vehicles, ground effect vehicle, autonomous WIG craft, stability, nonlinear control

## Abstract

Autonomous vehicles are nowadays one of the most important technologies that will be incorporated to every day life in the next few years. One of the most promising kind of vehicles in terms of efficiency and sustainability are those known as Wing-in-Ground crafts, or WIG crafts, a family of vehicles that seize the proximity of ground to achieve a flight with low drag and high lift. However, this kind of crafts lacks of a sound theory of flight that can lead to robust control solutions that guarantees safe autonomous operation in all the cruising phases.In this paper we address the problem of controlling a WIG craft in different scenarios and using different control strategies in order to compare their performance. The tested scenarios include obstacle avoidance by fly over and recovering from a random disturbance in vehicle attitude. MPC (Model Predictive Control) is tested on the complete nonlinear model, while PID, used as baseline controller, LQR (Linear Quadratic Regulator) and adaptive LQR are tested on top of a partial feedback linearization. Results show that LQR has got the best overall performance, although it is seen that different design specifications could lead to the selection of one controller or another.

## 1. Introduction

Autonomous transport systems and networks are rapidly becoming one of the key factors in order to achieve a true sustainable development, given its impact on environment, society and economy, and its core role in many other sectors. Therefore, the conception, design and implementation of new transport means and strategies based on them, giving answers to the increasing complexity that the relationships among those three aspects of human activity raise are crucial to fulfill the present and future needs of any kind of mobility. In this context, the development of autonomous transport vehicles that bring together speed, flexibility and energy efficiency is of critical importance.

Apart from Unmanned Ground Vehicles (UGVs), particularly self-driving cars, and Unmanned Aerial Vehicles (UAVs), which gather a huge attention from the scientific community from a long time on, one of the most promising research lines for exploring autonomous transportation systems is water, both in Unmanned Underwater Vehicles (UUV) and Unmanned Surface Vehicles (USV), given the special features of this medium. In fact, already in 2003 the European Commission established as priority strategy the development of “Motorways of the Sea” [1], ranked with the same importance as rail based or road based transport. Putting together both concepts, sustainability and autonomous water transport, the idea of designing transport networks based on water vehicles joining a convenient speed with a high energy efficiency or, even, zero emissions arise. One of the most promising ways to achieve all these objectives are vehicles that operate in ground effect [2].

Wing-in-ground (WIG) vehicles started to be systematically studied in the beginning of the 1960s of 20th century [3] in the Soviet Union by the team led by engineer Rostislav Alexeiev. During decades they were working in their development due to their potential as a tactic weapons and troops carrier, given their speed of movement over inner waters, and their huge lift power.

Studies in this field were based upon the observation of the fact that, for a fast vessel it is mandatory to reduce the hydrodynamic resistance acting on the vessel body. Moreover, this resistance grows quickly as the vessel increases its speed. Therefore, in order to reduce it the vessel must be kept away from the water surface as much as possible [3]. Henceforth, bodies were optimized in this sense, till eventually hydrofoil was introduced as a breakthrough directed to detach the vessel body from the water surface. Hydrofoils reduce significantly hydrodynamic resistance, since the contact of the vessel with water is limited to a minimum lifting surface, strongly improving sailing performance.

Nevertheless, there still exists a minimum contact between vessel and water. Therefore, the last step towards the complete detachment of body and water is removing the hydrofoil. This can be achieved by means of the ground effect, seizing the extra lift that the proximity of a rigid surface generates in an aerodynamic plane. In this way, and keeping the idea of “vessel”, the transformation from a traditional body to a shape that more clearly resembles a plane is accomplished. Nevertheless, and this is very important in operational terms, WIG crafts are still qualified as “vessels” in our regulatory frame, as they don’t reach significant heights over the water surface; given this qualification, it is not necessary to certify a WIG craft with the aeronautic authorities, making much simpler the requirements for setting up and operate a line.

**Figure 1:**
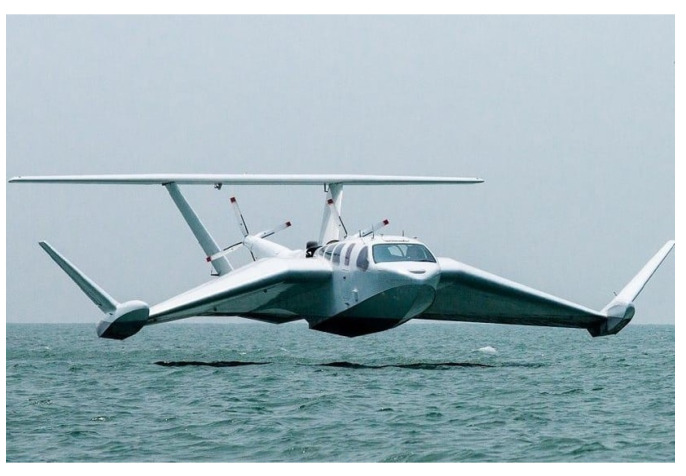
AirFish 8 WIG craft during cruising (WigetWorks).

The Soviet Union and afterwards Russia discontinued the development program, due to the technical and operational difficulties these vehicles present and the growing interest in nuclear submarines, and nowadays there are no meaningful efforts to bring their use back to regular and massive transport of people and/or goods, but isolated initiatives as, for instance, the company WigetWorks which is at present carrying out preliminary operation tests in Singapore; their craft, named AirFish 8 can be seen in Figure 1.

In terms of developing a fully autonomous USV based on this technology, efforts reported so far are sparse, and no sound research line for deploying a feasible autonomous WIG craft is on, to authors’ best knowledge, leaving a vast niche for exploring the possibilities of this kind of vessels in terms of autonomous operation. Moreover, given the advances in computation and specifically in CFD (Computational Fluid Dynamics), simulation and embedded computing, as well as control theory, authors consider that technology has developed to a state where the design and implementation of an autonomous WIG craft if possible, in such a way eventually this could be a feasible transport alternative.

In this article, we tackle the problem of automatic control for an autonomous WIG during the ground effect flight. Different control strategies are tested, taking into account two critical situations: obstacle avoidance by fly over and recovering heading after a disturbance, for instance a wind gust impact, or a sense and avoid maneuver. The results of the three strategies are compared in order to gain understanding on how to come closer to a valid, robust and safe solution.

The rest of the paper is organized as follows: Section 2 describes the previous works related to the study of ground effect, and the stability and control of WIG crafts. Section 3 deals with the dynamic model of a WIG craft, which is the starting point to study model-based control solutions. It also introduces the control strategies we have tested against that model; first, a non linear MPC (Model Predictive Control) is tested in the aforementioned representative manoeuvres of the WIG Craft. After that, two different versions of LQR (Linear Quadratic Regulator) on top of an input-output feedback linearization are tested under the same conditions; one of them represents a fixed approximation of the non-cancelled non linearities of the model, whilst the other one is an adaptive version, close to the concept of self tuning regulators, a well know Adaptive Control strategy. Finally, Section 4 presents the conclusions to the overall work and our expectations for the near future.

## 2. Previous Work

Ground effect is known, at least at an intuitive level, from the very beginning of aviation. It can be said that it has been exploited even in the early flights by the Wright Brothers, who flew long distances at a very low height seizing the extra lift force that ground effect provide to an aircraft [3].

Although some interesting theoretical explorations of this effect were accomplished during the 1920s [4], it was not till the early 1930s that Toivo Kaario came up with the idea of the specific development of a vehicle that makes use of maavaikutusta (ground effect in Finnish) for its displacement [5]. His idea was to use the ground effect to build vehicles that could glide on ice and/or calm water. However, the great interest on aeronautics for developing passenger flights put Kario’s studies apart. It was not till the early 1960s that Rostislav Alexeiev boosted the study of the экранный эффект (ground effect in Russian), starting a huge military effort that reached its summit with the Кoрабль-макет (Naval Ship Prototype in Russian), known as the ’Caspian Sea Monster’ after it was unveiled during USA spy flights in 1967. This ship weighted almost 550 tons and was able to carry up to 900 troopers at a cruise speed of 430 kph [3]. The program was continued till the 1990s, when it decayed. Nevertheless, efforts to get these ships in service were not completely abandoned by the scientific community, as different initiatives to get WIG vehicles in service have been reported.

In terms of technical contributions after Alexeiev’s program, there has been some advances in modelling and control of these ships. Efforts to model ground effect have been accomplished in order to establish how it influences the behaviour of air crafts as they are close to the ground in take off and landing manoeuvres. In this way ESDU (Engineering Sciences Data Unit that belongs to IHS Markit) has emitted its report introducing corrections to aerodynamic coefficients when a wing is affected by ground effect [6].

Ground effect has been further studied in works such as [7], where the author features the aerodynamic behaviour of a wing working close to the ground in take-off and landing manoeuvres. In this work, a novel algorithm for aerodynamic featuring of wings using viscous continuous adjoint formulation is introduced, and its performance tested in this particular flight phase. After that, in [8] CFD simulation cycles where used to gain understanding on aerodynamics of small scale WIG ships, and flight test performed afterwards to validate those numerical results. In [9] authors present a thorough study of the aerodynamic behavior of a WIG craft by using CFD simulations, introducing some findings concerning the periodicity of the aerodynamic forces as the craft proceed on a wavy surface; in the same direction, in [10] authors generalize this study to three qualitative different surface undulation amplitudes to determine how they affect to the aerodynamic behaviour of the craft. Close to these works, in [11] authors explore the effects of varying the angle of attack when overflying a wavy ground. In [12], authors present a set of exact solutions for ground effect, which comprises different parameter regimes, offering new understanding on the mathematical structure of this effect. A study on the effect of lateral moments in banking maneuvers of a WIG craft is introduced in [13], unveiling relationships among these moments, the aspect ratio of the wing and the angle of attack.

One interesting field of study is the control of ground effect crafts. In fact, control is the main barrier in order to give the last step leading to commercial exploitation of ground effect crafts. In [14] a MIMO model to be used as the basis for further studies regarding control was introduced. It was shown how aerodynamics in strong ground effect affects the behaviour of the craft and how to translate it to a valid mathematical model that can be used for testing control strategies.

Tightly linked with the flight control problem, some studies have introduced the question of flight stability. For instance in [15] the author establishes relations between longitudinal stability of the craft and its aerodynamic design; the assumption is that the craft flies at constant speed, and one of the most important conclusions is that center of height must be ahead of the center of pitch, which in fact can be taken as a design principle for WIG crafts; as defined in [15], “the center of height is the point of application of a lift increment because of a change in flight altitude”. In [16] authors tackle the study of the effect of ground vicinity on the longitudinal stability of a WIG craft, analysing the effects of varying ground clearance on the vehicle dynamics. In [17] authors study the cross influence of different design parameters and the flight stability of a WIG craft. In [18] the author present a thorough study of flight dynamics of WIG crafts, followed by an analysis of performance and stability referred to different equilibrium conditions, making broad use of numerical calculations to study the equations of motion; in [19] authors introduce a method for computing unsteady aerodynamic derivatives using Reynolds-averaged Navier–Stokes simulations, and they use these results to study their effect on longitudinal stability. In [20] the author present a detailed study on the longitudinal stability of a standard NACA4412 airfoil, whose mean camber line has been previously modified giving it a partially flat lower surface to achieve an S-shaped profile in order to improve its height stability. A broader study is introduced in [21], where the authors not only focus on the longitudinal stability of the craft during the flight phase, but also in hydrodynamic stages of operation, that is including take-off and landing. In [22], authors take stability studies one step further, proposing three real and one virtual designs of WIG crafts with improved stability.

Regarding automatic control of WIG crafts, only a few preliminary studies can be found. However, precise control is a major issue for commercial exploitation of WIG crafts as an alternative to classic surface vessels for water transport [14]. One interesting work is presented in [23], where the author presents a complete modelling-testing-validation effort in order to approach to the problem of controlling a WIG craft. This work is based on classic linear approximations of the vessel in selected working points, but its value lies on the fact that it tackles the problem of automatic height control. In [24] the authors approach the control problem assuming that the WIG craft operates on a periodically wavy surface, which happens to be a mathematical model of a disturbed sea. In [25] authors present a comprehensive review on the research on stability control for WIG crafts.

## 3. WIG Craft Control Strategies

The simpler control case for a vessel is the seakeeping scenario, that is the cruising mode where heading, height and angle of attack are kept constant. This is the most common way of operating a vessel, since assuming no disturbances affect its attitude allows the vehicle to go from one point to another following a straight line, i.e., following the shortest path.

A promising control strategy based on the complete non-linear model will be used in first place, namely MPC (Model-based Predictive Control). MPC is a candidate for more demanding control tasks than seakeeping, given its high complexity, that makes it particularly suitable for WIG craft control. For instance, non-linear MPC is theoretically capable of obstacle avoidance by fly over. Proving so would allow us to use it for broader cases than steady state keeping, opening the possibility of planning and controlling much more complex manoeuvres. Such tasks are possible using the non-linear model, but would be hardly possible for simpler linear controllers, whose capabilities are by design limited by the range of applicability of the underlying linear approximation of the actual non-liner model.

After the MPC strategy, we have tested LQR (Linear Quadratic Regulator) and PID controllers on top of a feedback linearization, a strategy based upon cancelling non-linearities of the model, whether partially or totally, by means of a convenient state feedback. In our case, we have provided a feedback partial linearization, leaving some underlying non-linearities to deal with. In order to cope with them, two approaches will be tested: the first one is based on a single linear approximation that will be used during the whole experiment; in the second one, an adaptation mechanism that modifies the linear approximation as the working point evolves is introduced, which puts this strategy close to the Adaptive Control concept of self-tuning regulators [26].

For the LQR and PID implementations only direct inputs which correspond to the 6 DOFs of the vessel attitude, i.e., 3D position and Euler angles, are used. These are considered as solely acting along one direction, with no coupling effects. This is done to simplify the model enough in order to allow for a simpler formulation, but it is also a common practice in linear approximations for autonomous vehicles [27].

The main goal of this study is to evaluate both controllers performance for steady state cruising and fly over obstacle avoidance. The input expressions introduced in [18], broadly accepted in modelling WIG crafts, are quite complex since they introduce more coupling between the inputs, and put extra strain in the optimization process, so they are used solely with the non-linear MPC. For the MPC case the motor acts solely along $x_{b}$, and the surfaces, i.e., elevators, ailerons and rudders, as needed and possible for the demanded configuration. Their range of mobility is considered to be in $\left[-\frac{\pi }{2},\frac{\pi }{2}\right]$.

### 3.1. WIG Craft Equations of Motion

**Figure 2:**
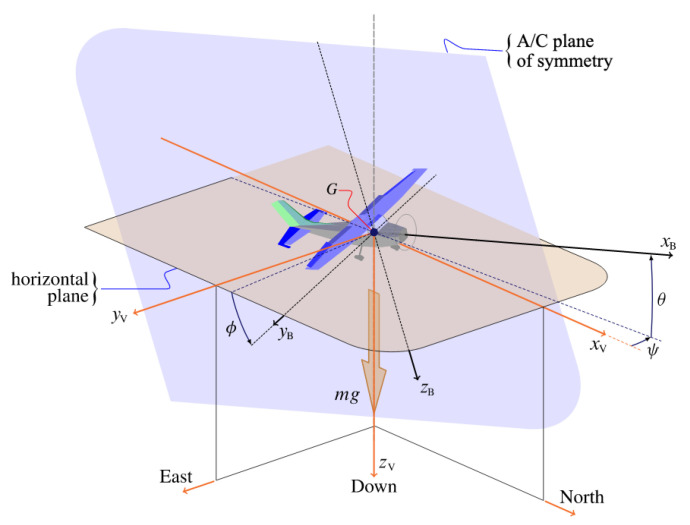
Reference systems. Courtesy of JSBSim Team.

The 6 DOFs (degrees of Freedom) modelling of WIG crafts has a strong empirical component, as the behaviours produced in this operating regime depend on the geometry of the vehicle. The vector form of the classic dynamic equations for a WIG craft, referred to the coordinate systems shown in Figure 2, can be presented in the following form [18].

(1) $$\left\{\begin{array}{c} \begin{array}{cc} \; & m\left({\dot{v}}_{b}+\omega \times v_{b}\right)=F^{e}\\ \; & J_{G}^{b}\dot{\omega }+\omega \times J_{G}^{b}\omega =M^{e}\\ \; & \dot{x_{i}}=Tv_{b}\\ \; & \dot{\Phi }=R\omega \end{array} \end{array}\right.$$

The first two equations represent the force and angular momenta equations in body frame respectively, and the other two the transformations to inertial frame with J as the inertia tensor. The vector ${\dot{x}}_{i}={\left[x,y,z\right]}^{T}$ represents the velocity with respect to the inertial frame, $x_{i}$ is the position vector, and the altitude *h* is taken into account by z=−h. The matrices T and R are transformation matrices used to rotate from the body referred frame to the inertial one. The components of the body referred linear and angular velocities are $v_{b}={\left[u,v,w\right]}^{⊺}$ and $\omega ={\left[p,q,r\right]}^{⊺}$. The external forces and torques are represented respectively by $F^{e}$ and $M^{e}$, which also contain aerodynamic terms. The vector Φ represent the Euler angles, called, in order roll, pitch and yaw and m is the mass of the WIG craft.

The expanded form of the forces equation is the following

(2) $$m\left(\begin{array}{c} \dot{u}+qw-rv\\ \dot{v}+ru-pw\\ \dot{w}+pv-qu \end{array}\right)=mg\left(\begin{array}{c} -S\theta \\ C\theta \;S\phi \\ C\theta \;C\phi \end{array}\right)+\frac{1}{2}\rho A{∥V_{a}∥}^{2}\left(\begin{array}{c} C_{x}\left(h\right)\\ C_{y}\left(h\right)\\ C_{z}\left(h\right) \end{array}\right)+\left(\begin{array}{c} F_{x}^{b}\\ F_{y}^{b}\\ F_{z}^{b} \end{array}\right)$$

where ρ stands for the density of air, Sθ=sinθ, Cθ=cosθ and, later on, Tθ=tanθ, *A* the reference surface, usually the wing one, ρ is the fluid density, air in this case, ${\left[C_{x}\;C_{y}\;C_{z}\right]}^{⊺}$ the body referred aerodynamic coefficients which in this case are strongly dependent on the altitude *h* from the water surface, and finally the last term represents the external forces, in this case the thrust.

WIG crafts are usually symmetric with respect to a vertical plane so the inertia tensor is of the form

(3) $$J_{G}^{b}=\left(\begin{array}{ccc} A & 0 & -E\\ 0 & B & 0\\ -E & 0 & C \end{array}\right)$$

The expanded angular momenta equation on body frame is

(4) $$\begin{array}{ccc} \; & \left(\begin{array}{c} A\dot{p}-E\dot{r}+rq\left(C-B\right)-Epq\\ B\dot{q}+rp\left(A-C\right)+E\left(p^{2}-r^{2}\right)\\ -E\dot{p}+C\dot{r}+pq\left(B-A\right)+Erq \end{array}\right)=\; & \frac{1}{2}\rho A\ell V_{a}^{2}\left(\begin{array}{c} C_{l}\left(h\right)\\ C_{m}\left(h\right)\\ C_{n}\left(h\right) \end{array}\right)+\left(\begin{array}{c} M_{F_{x}^{b}}\\ M_{F_{y}^{b}}\\ M_{F_{z}^{b}} \end{array}\right) \end{array}$$

The last two equations of (1) are the transformations between the quantities in body frame and the vehicle-carried frame. The following represents the relationship between the derivatives of Euler angles, which are roll, pitch and yaw and the angular velocities in body frame p,q and *r*.

(5) $$\left(\begin{array}{c} \dot{\phi }\\ \dot{\theta }\\ \dot{\psi } \end{array}\right)=\left(\begin{array}{ccc} 1 & S\phi \;T\theta & -S\theta \\ 0 & C\phi & -S\phi \\ 0 & \frac{S\phi }{C\theta } & \frac{C\phi }{C\theta } \end{array}\right)\left(\begin{array}{c} p\\ q\\ r \end{array}\right)$$

This allows us to find the relative orientation of body frame with respect to the vehicle-carried frame, obtaining the angular configuration of the vehicle.

The rotation matrix in the last equation of (1) is

(6) $$T=\left(\begin{array}{ccc} C\theta \;C\psi & S\theta \;S\phi \;C\psi -S\psi \;C\phi & C\psi \;S\theta \;C\phi +S\phi \;S\psi \\ S\psi \;C\theta & S\theta \;S\phi \;S\psi +C\psi \;C\phi & S\theta \;C\phi \;S\psi -S\phi \;C\psi \\ -S\theta & C\theta \;S\phi & C\theta \;C\phi \end{array}\right)$$

Therefore

(7) $$V_{i}=TV_{b}$$

Once the model has been completed, it is worth noting that no matter how many of the 6 DOFs of the vessel are to be controlled, underlying nonlinearities cause that closing a loop on one influences the rest of them. For that reason, the complete model will be considered through our experiments.

**Figure 3:**
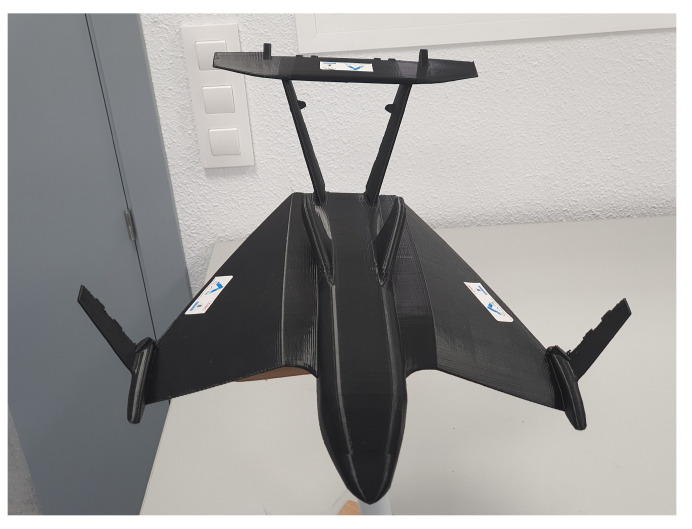
Physical model for wind tunnel and motion capture experiments.

For our experiments we have considered a very similar design to WigetWorks AirFish 8, so for all the following simulations and studies the aerodynamic behaviour has been featured using its morphology. In Figure 3, a physical model ready for wind tunnel and motion capture experiments can be seen.

### 3.2. Non-Linear Model Predictive Control

This first control strategy that has been analyzed is a nonlinear MPC (Model Predictive Control), that allows one to use the previously obtained nonlinear model model; MPC is an optimal control strategy that can be applied both to linear and nonlinear dynamics [28]. The used environment is do-mpc python library [29], a toolbox for non-linear robust MPC based in CasADI [30] and IPOPT.

Model predictive control considers an objective function, which is used to determine the optimal inputs, and a reference model, which is used to predict the system behaviour. In this case the simulated model coincides with the reference model. The tuning, and therefore the behaviour, of the controller is given by the objective function, which has the general form:

(8) $$J\left(x,u\right)=\sum_{k=0}^{N}\left(\underset{\mathrm{stage}\mathrm{cost}}{\underset{︸}{l(x_{k},u_{k},p_{tv,k})}}+\underset{\mathrm{input}\mathrm{cost}}{\underset{︸}{\Delta u_{k}^{T}R\Delta u_{k})}}\right)$$

where *N* is the prediction horizon, $x_{k}$ the states, $u_{k}$ the inputs and $p_{tv,k}$ are the time varying parameters. Each term of the cost function *J* represent, respectively: stage cost represents the instantaneous expense evaluated at each time instant according to a custom function, which also contains the final states values; input cost solely focuses on inputs, quantifying the relative cost of each input through the weight matrix *R*.

This objective function is utilized by the optimizer to compute the optimal input. This is done by using the model fed to the controller to predict the evolution of the system, stage by stage, from the present instant until the instant *N*, which is also a parameter of the controller.

A relative equilibrium position is sought for the reference values of $v_{in,x}=30\;\frac{m}{s}$ and altitude=1m. The pitch value is considered as a bounded state rather than a requirement. Therefore, even though the pitch belongs to the equilibrium states, it affects the lift force through the dependency upon it, so it is considered a free parameter let to be chosen by the optimizer. An equilibrium position at the given altitude and speed is found through the control loop itself giving the following results:

(9) $$\begin{array}{cc} \; & v_{i,x}=30\;\frac{m}{s}\\ \; & \theta =0.045\;\mathrm{rad}\\ \; & h=1\;m\\ \; & F_{b,x}=432\;N\\ \; & M_{b,y}=-56\;\mathrm{Nm} \end{array}$$

In the following, we present the results of the simulations carried out on controlling two different maneuvers. The first control scenario is obstacle avoidance by fly over, starting from the equilibrium position. Reaching a final steady state on altitude is the control objective.

#### 3.2.1. Altitude Variation Using MPC

**Figure 4:**
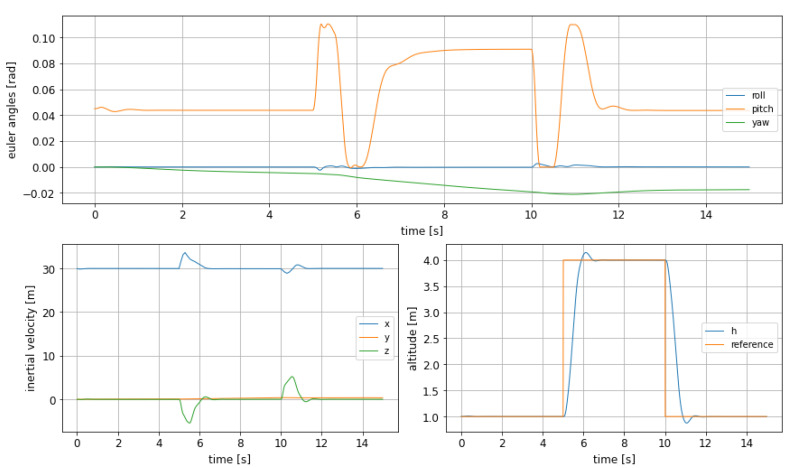
Euler angles (**top**), inertial velocities (**bottom left**) and altitude change (**bottom right**) with MPC, using the 3 control surfaces from [18].

Starting from an altitude of 1m, the system is given a new reference altitude of 4m, which is relatively a big step (see Figure 4). The parameters selected to express the performance of the controller are rise time ($t_{r}$), settling time ($t_{s}$), and overshoot.

Given the different order of magnitude of the two type of inputs the weights are assigned to even them out. They are chosen empirically, and the general principle is to even the different orders of magnitude, in order to obtain a smooth behaviour and steady state for all the states. In this way a weight of 100 is given the control surfaces and a weight of 1 is given to motor. The cost function is

(10) $$J=\int_{0}^{t_{h}}\left(10,000\phi^{2}+1000\psi^{2}+0.5{\left(v_{1}^{i}-v_{1,fin}^{i}\right)}^{2}+{v_{2}^{i}}^{2}+50{\left(h-h_{fin}\right)}^{2}\right)d\tau$$

In Table 1 the characteristic values of the WIG craft response as read from the graphics in Figure 4 can be seen: the rise time is short, at the cost of significant oscillations in pitch; nevertheless, as this experiment has been conducted to evaluate the system behaviour in altitude, the results are satisfactory, as it is demonstrated that the craft can change its altitude and fly over an obstacle in an agile way.

The yaw angle shows what seems to be a steady state error (see Figure 4), which however does not generate a big deviation in the inertial speed. This can be explained by the small weight associated to this variable in the objective function, so this error is being corrected too slowly by the controller.

For the purpose of correcting the fast oscillations in the pitch, a different tuning has been tested, since this could be a major issue as it could affect the comfort and safety of the passengers on board.

The following modifications have been performed to the objective function and other parameters. First of all, a slightly different approach that consists in defining a function that relates the altitude equilibrium value to a pitch angle, for a given speed is used. Doing so the controller can be set for the final value of the pitch based on the final altitude. This will make the pitch angle oscillate less, leading to a smoother dynamic.

A function relating these variables can be obtained through the static equation of vertical motion in the inertial frame. The velocity alongside $x_{b}$, which is the first axis of the body referred frame pointing toward the nose, also influences the lift force, and in this case it is considered as fixed and having a value of $30\;\frac{m}{s}$, which is the steady state cruising speed. By fixing the speed, the lift force now solely depends on the angle θ and the altitude *h*.

(11) $$F_{lift}\left(\theta ,h\right)-mg=m\ddot{h}$$

By imposing $\ddot{h}=0$, which represents a condition of vertical equilibrium alongside the axis, a function of θ and *h* is obtained.

Using this data a target value can be computed for the final pitch given the final altitude. In this case, setting the final altitude at 4m as before, the result is θ=0.10rad.

Including this information in the objective function it becomes

(12) $$J=\int_{0}^{t_{h}}\left(10,000\left(\phi^{2}+{\left(\theta -\theta_{f}\right)}^{2}+0.1\psi^{2}\right)+0.5{\left(v_{1}^{i}-v_{1,fin}^{i}\right)}^{2}+{v_{2}^{i}}^{2}+50{\left(h-h_{fin}\right)}^{2}\right)d\tau$$

In addition to this, the bounds on the state values modified with respect to the previous cases: now they are bounded between [0,0.110], while in the last experiment they were limited to the interval [−0.350,0.350].

**Figure 5:**
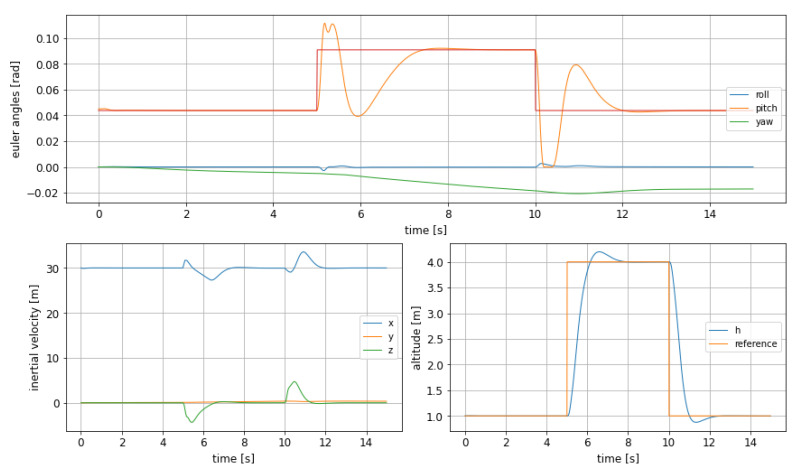
Euler angles (**top**), inertial velocities (**bottom left**) and altitude change (**bottom right**) with MPC, adding bounds on pitch and using the corrected objective function in (12).

The dynamic shows the foreseen improvement, at the cost of slower performances: in Figure 5 it can be observed how the pitch shows a much smaller oscillation, solving the issue formerly detected, but keeping the rise time still short enough to allow obstacle avoidance in an agile way if needed. However, the effect on altitude overshoot is much smaller, as it decreases from 7.5% to only 4.9%, being, however, smoother as well.

Nevertheless, it must be highlighted that the difference in terms of response between both cases is not negligible as the rise time more than doubles compared to the previous case, increasing from 0.45s to 1.16s, as can be read in Table 2. This fact would impact the choice of a controller version over the other, depending, for instance, on the time in advance an obstacle can be detected.

**Figure 6:**
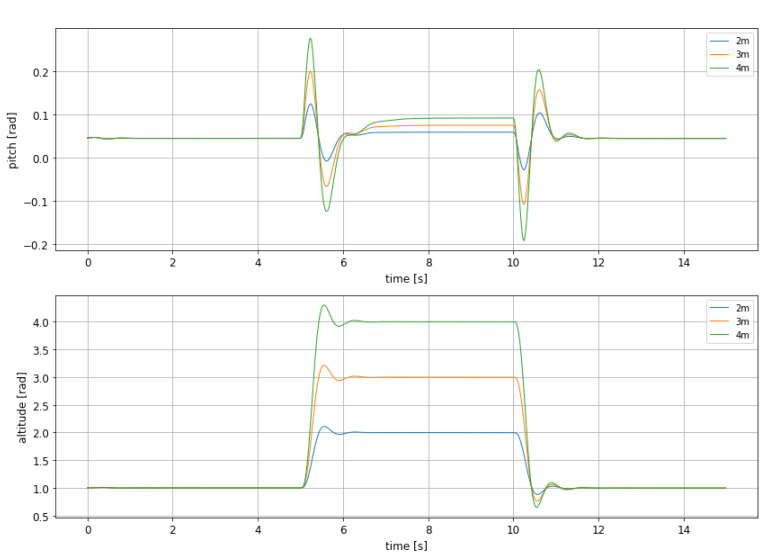
Comparison between the altitude and pitch dynamic for the non-linear MPC with different rise altitudes.

Taking into account that this simulation has been run in order to evaluate the performance of this controller in a hopping maneuver, where the craft has to fly over the obstacle, it seems natural to choose the controller that shortens the altitude change time as much as possible, i.e., leaving pitch unbounded. with this idea in mind, new simulations were run, assuming different final altitudes as not all the obstacles has the same height and therefore is not always necessary to make the same maneuver. In Figure 6 the evolution of pitch and altitude for different altitude changes can be seen.

It is important to highlight that for evaluating the performance of this controller undershoot is even more important than overshoot, as it reflects how close to the water surface the craft gets during the obstacle avoidance. Needless to say that touching the water can lead to a sudden and critical change of the craft dynamics, jeopardizing its integrity. Therefore, both overshoot and undershoot has been measured an depicted in Table 3 and Table 4.

From both tables it can be seen that overshoot and undershoot take acceptable values, as they reflect a smooth behavior, mostly during rise, and a safe descent to the cruise altitude, as undershoot takes the craft to a height which is far enough from water surface.

#### 3.2.2. Altitude Change with Constraints on the Control Surfaces Mobility

**Figure 7:**
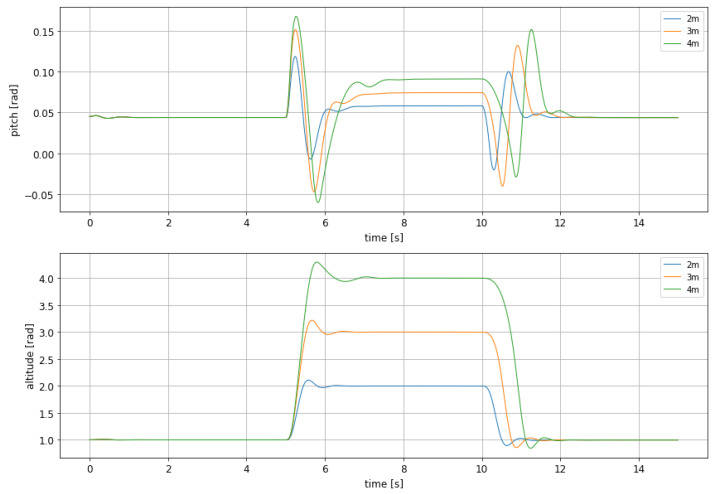
Comparison between the altitude and pitch dynamic for the non-linear MPC with different rise altitudes with constrained control surfaces.

The previous simulations have been carried out considering a rather permissive input surfaces operating range. This has been done in order to be able to compare the MPC controller, that is designed to include bounds on states and inputs, with the other controllers, that hardly allow saturation on the behaviour of the system variables. For the sake of completion the same simulations as those shown in Figure 6, whose evaluation parameters are shown in the Table 3 and Table 4, have been performed using actuator saturation: the action made by the control surfaces is always within $\left[-15^{\circ },15^{\circ }\right]$. Results are shown in Figure 7.

The figure clearly shows that it is harder for the controller to set the output at the right value as target altitude becomes higher. This behaviour is even clearer in the descent phase, where controlling the WIG craft is more difficult. Table 5 and Table 6 show a significant change in the evaluation parameters compared the previous case, represented by Table 3 and Table 4.

#### 3.2.3. Recovering from a Disturbed Attitude

This control scenario using the MPC shows a borderline feasible case for situations such as a significant displacement from cruising conditions that needs to be restored as soon as possible. The MPC has shown great performances in the previous cases, so good results are expected here too.

The initial attitude is chosen to be

(13) $$\begin{array}{c} \left\{\begin{array}{c} \phi_{0}=11.46^{\circ }\\ \theta_{0}=6.01^{\circ }\\ \psi_{0}=-11.46^{\circ }\\ h=1\;m \end{array}\right. \end{array}$$

The objective function is set back to the expression defined in (10).

**Figure 8:**
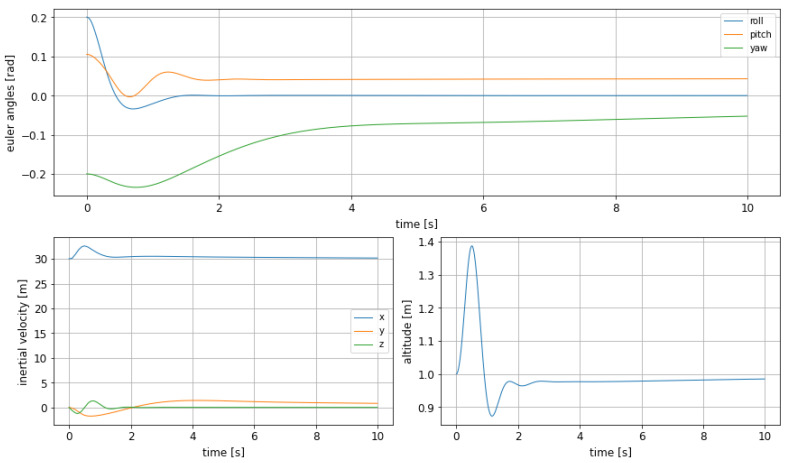
Euler angles (**top**), inertial velocities (**bottom left**) and altitude change (**bottom right**) in the experiment of steady state recovering from a random unsteady attitude.

The evaluation parameters include now the undershoot, that has already been considered in the previous section, which measures how low, percentage-wise, the altitude goes before reaching the prefixed steady state. This effect can be clearly seen in Figure 8, in the bottom right graphic, where the evolution of altitude is depicted. The precise values of undershoot and the other response characteristics can be seen in Table 7.

As a conclusion it can be stated that in this case it is important that the altitude does not reach too low values, which would be a safety concern as could increase the risk of an unwanted contact between the craft and the water; altitude appears to be quite well bounded in terms of oscillations. In the top part of the figure it can be seen, as well, that the Euler angles are well bounded, and have a soft transition to the steady state value.

Moreover, it is worth to note that a combination undershoot and high roll values could lead to wing tips to touch the water surface. However, given the maximum roll values we have observed, the minimum height and the wingspan of our model, this risky situation is always far from arising. The same can be stated for extreme pitch values: neither the nose nor the tail of the craft would touch the water with the values that are reached during our experiments.

### 3.3. Feedback Linearization

Feedback lnearization is a control technique designed for transforming totally or partially a nonlinear system in a linear equivalent, by means of a state feedback which cancels available nonlinearities of the dynamic model [31].

First of all, let us recall the dynamical equations general form

(14) $$\left\{\begin{array}{c} \begin{array}{cc} \; & m\left({\dot{v}}_{b}+\omega \times v_{b}\right)=F_{e}\\ \; & J_{G}^{b}\dot{\omega }+\omega \times J_{G}^{b}\omega =M_{e}\\ \; & {\dot{x}}_{i}=Tv_{b}\\ \; & \dot{\Phi }=R\omega \end{array} \end{array}\right.$$

The principle of input-output linearization is to choose the inputs in order to obtain a linear relationship between these and the output. This represents a less coomplicated and more attainable kind of linearization compared to input-state, which would lead to a fully linear system.

Unfortunately, given the existing relationship between the accelerations and body frame and the velocities in inertial frame, it is not possible to obtain an input-state linearization, because of the transformations in the third and fourth equations of (1). Therefore, the inputs are chosen in order to cancel the non linearities in the body-referred forces and angular momenta equations.

The steps required to achieve this objetive are the following.

The body referred equation can be simply linearized, as is shown for the first angular velocity equation from (14).

Given the expanded form (4) of the first component of angular velocity in body frame

(15) $$A\dot{p}=E\dot{r}-rq\left(C-B\right)+Epq+M_{aer,x}+M_{mot,x}$$

where $M_{mot,x}$ is the input torque from the motor. By choosing it as

(16) $$M_{mot,x}=-A\left(\left(E\dot{r}-rq\left(C-B\right)+Epq\right)-M_{aer,x}+M_{mot,x}^{\prime }\right)$$

where the last term represents an independent input, the equation (15) becomes

(17) $$\dot{p}=M_{mot,x}^{\prime }$$

Applying the same principle to all other body referred acceleration equations the result is

(18) $$\begin{array}{c} \left\{\begin{array}{c} \dot{u}=F_{mot,1}^{\prime }\\ \dot{v}=F_{mot,2}^{\prime }\\ \dot{w}=F_{mot,3}^{\prime } \end{array}\right.\;\left\{\begin{array}{c} \dot{p}=M_{mot,1}^{\prime }\\ \dot{q}=M_{mot,2}^{\prime }\\ \dot{r}=M_{mot,3}^{\prime } \end{array}\right. \end{array}$$

The resulting equations are perfectly linear and decoupled, with each input acting on one state only, without any coupling effects present.

However, coupling is still present in the inertial velocities, in particular in the third component, that is the part of the linearized model to be taken into account.

Compared to regular series expansion, the big advantage of feedback linearization is that it is valid in the full range of values of the involved variables, and not only in the neighbourhood of a single chosen point. Of course the quality of the process is subject to the sensors performances used in the real case to compute the values of the states to be fed back. Right now such considerations are not going to be made and the simulations will not include this effect, given that there are no information about such process apart from a general perspective on the alleged problems that can cause.

#### 3.3.1. LQR Controller on Top of Mixed Linearized Model

Given a linear system of the form

(19) $$\dot{x}=Ax+Bu$$

The LQR strategy consists of considering a quadratic cost function

(20) $$J=\int_{0}^{\infty }\left(x^{T}Qx+u^{T}Ru+2x^{T}Nu\right)dt$$

The feedback control law that minimizes this cost is

(21) u=−Kx

where the feedback is given by

(22) $$K=R^{-1}\left(B^{T}P\left(t\right)+N^{T}\right)$$

*P* is found by solving the continuous time Riccati differential equation

(23) $$A^{T}P\left(t\right)+P\left(t\right)A-\left(P(t)B+N\right)R^{-1}\left(B^{T}P\left(t\right)+N^{T}\right)+Q=-\dot{P}\left(t\right)$$

As shown above it is not possible to linearize the model in inertial velocities and inputs, thus in altitude, using feedback since the non-linear relations do not allow so. Henceforth, another layer of linearization is needed for a linear controller to be applied.

One of the ways the feedback linearization can be exploited is to apply it to the body-referred Equation (1), as shown above and linearize the remaning part of the model to build an LQR controller.

Given the chosen states amongst the available ones

(24) $$x={\left[\begin{array}{cccccccccc} p & q & r & u & v & w & \phi & \theta & \psi & h \end{array}\right]}^{T}$$

where the last state is the altitude, the matrix *A* assumes the form
(25)
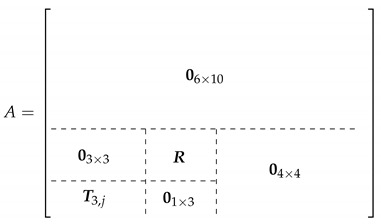

where R and $T_{3,j}$ are respectively the transformation from angular velocities in body frame to Euler angles and the third row of the rotation matrix for the linear velocities.

The chosen equilibrium position in terms of Euler angles, in radians, is the same as (9)

(26) $$\Phi =\left[\begin{array}{ccc} 0 & 0.045 & 0 \end{array}\right]$$

The *B* matrix is simply

(27) $$B=\left[\begin{array}{cccccc} 1 & 0 & 0 & 0 & 0 & 0\\ 0 & 1 & 0 & 0 & 0 & 0\\ 0 & 0 & 1 & 0 & 0 & 0\\ 0 & 0 & 0 & 1 & 0 & 0\\ 0 & 0 & 0 & 0 & 1 & 0\\ 0 & 0 & 0 & 0 & 0 & 1\\ 0 & 0 & 0 & 0 & 0 & 0\\ 0 & 0 & 0 & 0 & 0 & 0\\ 0 & 0 & 0 & 0 & 0 & 0\\ 0 & 0 & 0 & 0 & 0 & 0 \end{array}\right]$$

To evaluate its performance, a disturbed initial attitude is randomly chosen. The initial position is the same as before for comparison purposes (Equation (13)).

It is important to highlight that the equilibrium position is valid as long as the Euler angles are close to the linearization point, but the other states do not interfere with the linearization, since the feedback cancels those effects.

This kind of controller acts quite differently compared to the MPC, because the model is simplified and the full dynamic is not visible. While the MPC mainly relies on a change in θ in order to generate enough lift force for the altitude to change, in conjunction to a proper arrangement of the surfaces so that the resulting force is mainly along $z_{i}$, the model used for LQR does not include such part of the dynamic. This leads to the speculation that such controller is suitable for cruising, since the required control actions are limited compared to manoeuvring, but might not be efficient for other more sophisticated purposes. Additionally, given that the LQR does not allow for constrains as the MPC does, the range of effectiveness has to be evaluated carefully.

**Figure 9:**
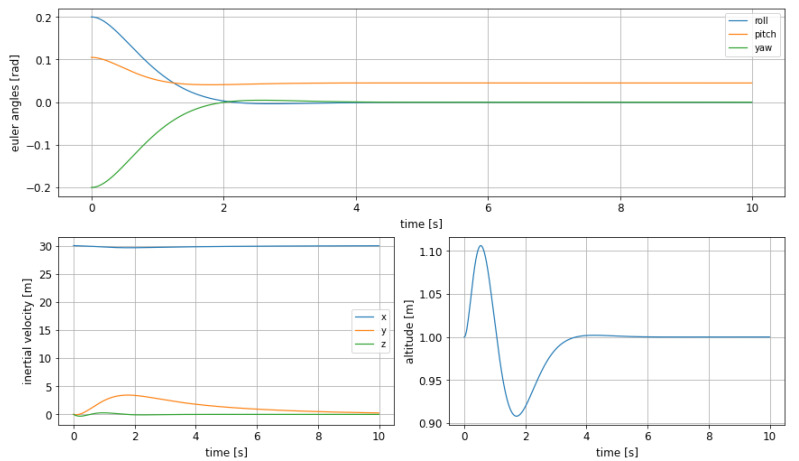
Euler angles (**top**), inertial velocities (**bottom left**) and altitude change (**bottom right**) using LQR control on linearized system, starting from disturbed attitude.

Figure 9 shows how the error compared to the cruising configuration is small, but it is still present on every component of the states.

The used weighting matrix *Q* is

(28) $$Q=\mathrm{diag}{\left[\begin{array}{cccccccccc} 0.1 & 0.1 & 0.1 & 1 & 1 & 1 & 10 & 10 & 10 & 100 \end{array}\right]}^{T}$$

The weights are ordered as the states in (24).

As expected the control action that is mainly used is the force along $z_{b}$, that generates a variation in altitude, which in turn presents a strong overshoot. It has been also observed during the experiments how a lateral dumped non-oscillating force is required along $y_{i}$ to correct the lateral velocity component generated by the initial non-straight direction of the ekranoplan.

As a general conclusion, it can be stated that the performance of the controller on Euler angles is smooth and acceptable.

However, when we put the LQR to undertake the task of controlling altitude during a hopping maneuver, results where far from satisfactory, yielding no acceptable results. Our interpretation is that the controller is not able to cope with the underlying non linearities as variable values get apart from the initial equilibrium point, that used for their linearization. Under this vision, it was decided to move to an adaptive version of LQR, in which the system dynamics matrix is recomputed over time in order to update the linear approximation as the system variables’ values evolve. In this way, this controller assume the same working principle as auto-tuning Adaptive Control, described in [26].

#### 3.3.2. Updating LQR over Feedback Linearized Model for Altitude Change

The LQR controller is built using the matrices of the linearized system, which constitute an approximation. The range of motion can reach points too far away from the linearization one, causing errors and bringing unreliability to the behaviour.

Given that the *A* matrix is of the form (25), its linearization can be recomputed during the simulation loop for the updated position. This allows us to keep the controller closer to the linearization point at the cost of solving again for each time instant the algebraic Riccati equation.

Doing so the issue with non-linear transformations in (1) can be tackled and reduced, extending the possible use cases for this controller.

This kind of adaptiveness can be tuned according to the requirements. The first and most important could be the computational constrain, which, as said previously when referring to the MPC, has to be assessed separately, since another level of simulation is needed.

Then, the simulation for altitude change was repeated using this adaptive version of LQR. The initial position is the usual equilibrium position for h=1m (9) and the sought position is h=4m, which requires a pitch angles of about θ=0.10rad.

**Figure 10:**
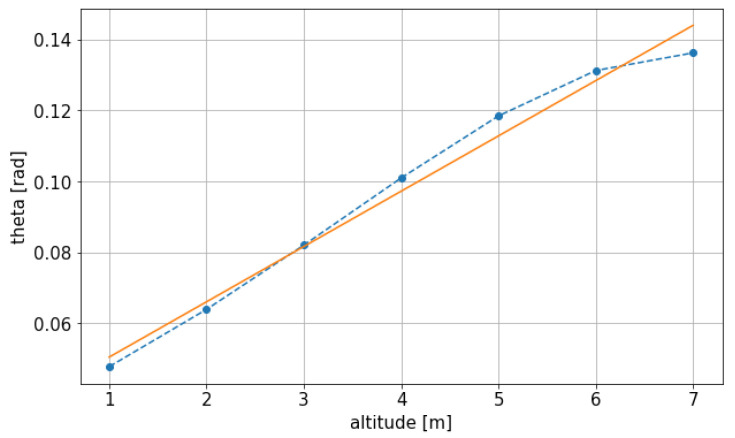
Obtained function, interpolated and scattered values for the vertical equilibrium position.

This pitch value is computed through Figure 10 and is manually set as the final target, as the is needed for the present case. It could be set avoided and left to the initial values of θ=0.045rad, given that the equations do not require an equilibrium position to be found. Instead it is done manually given the experience with the MPC, which reached a high enough pitch in order to save input cost increasing the pitch.

This could even be a feasibility matter, as the surfaces might not generate enough lift for the initial pitch value to be enough. Apart from the feasibility, it is clear that the pitch change makes the control process more efficient and requires less effort, so the behaviour of the MPC is imitated by manually setting the values.

The feedback linearization hides the cost of the linearization, so this other layer of control is implemented to limit it, confident that it will be realistically useful in real control scenarios once this particular control strategy will be applied in the real case.

The used weight matrix *Q* is

(29) $$Q=\mathrm{diag}{\left[\begin{array}{cccccccccc} 0.1 & 0.1 & 0.1 & 1 & 1 & 1 & 10 & 10 & 10 & 100 \end{array}\right]}^{T}$$

**Figure 11:**
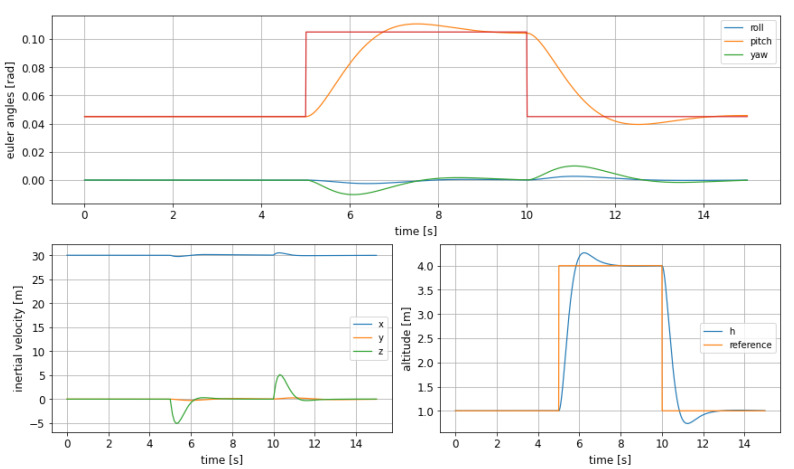
Euler angles (**top**), inertial velocities (**bottom left**) and altitude change (**bottom right**) in altitude change using LQR.

As can be seen in Figure 11, the controller almost does not cause overshoot in pitch and the overall transition is quite smooth. It is worth to note that the weight that has been set for the altitude state is set to 100, 1000 times more than the weight of the velocity components and 10 times more than the angles one. The characteristic values of the altitude behaviour can be read in Table 8, where it can be observed that the smooth evolution of pitch is reflected in the low overshoot of altitude.

Comparing the evaluation parameters the MPC is better if the objective is obstacle avoidance in the shortest possible time, but the LQR on the feedback linearised model might provide a smoother transition between two different cruising conditions when celerity is not crucial.

**Figure 12:**
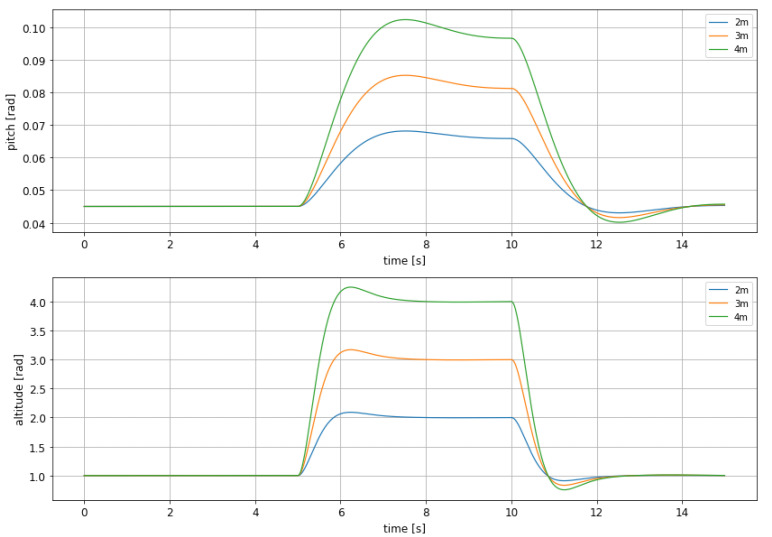
Comparison between the altitude and pitch dynamic for the updating LQR with different rise altitudes.

Being the case that adaptive LQR has yielded a promising result in this initial simulation, the same batch that was used for evaluating MPC in different altitude changes has been tested against this new controller. Results can be seen in Figure 12.

In Table 9 and Table 10 the values of height overshoot and undershoot, respectively, can be observed, as measured in curves depicted in Figure 12.

The overshoot and undershoot values depicted in these tables confirm what could be observed during the initial simulation: a smoother and slower behavior, which makes this controller more suitable when comfort during maneuvering is an issue. Anyway, once again is shown that undershoot reach safe values, as the craft keeps a safe clearance at any moment.

#### 3.3.3. Updating LQR for Recovery from Disturbed Attitude

This initial position is the same as the previous case shown using the regular LQR controller, in order to later compare the results. Since the LQR updates itself every cycle, the distance from the linearization position is not, in theory, a problem at all. The weights are the same as (29).

**Figure 13:**
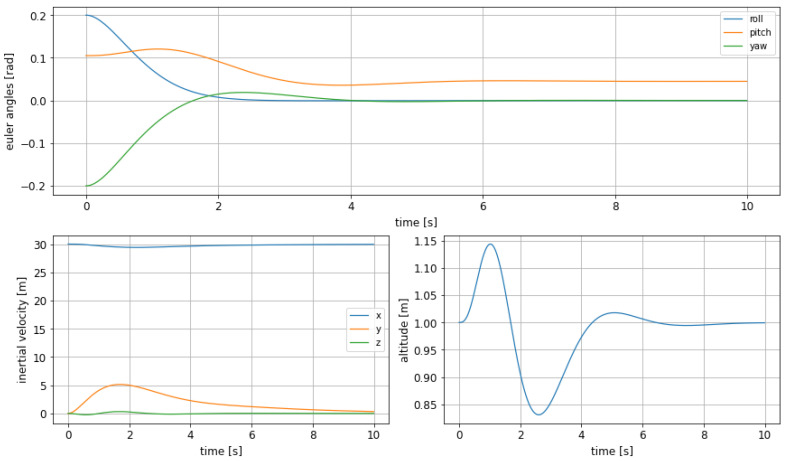
LQR controller for recovery from disturbed attitude.

In Figure 13 it can be observed, as the most salient feature, how the overshoot in altitude is much bigger than for the rest of controllers. This comes together with the fact that the settling time is much slower as well; putting both facts side by side it can be stated that the tuning of this particular LQR has caused a much slower reaction in the control system, allowing much bigger oscillations as it trends to react in a smoother way.

#### 3.3.4. PID Baseline Controller

A set of PID controllers is deployed for the sake of having a well known baseline to evaluate the results achieved with the LQR controller. The case under analysis is the same as Section 3.2.3.

**Figure 14:**
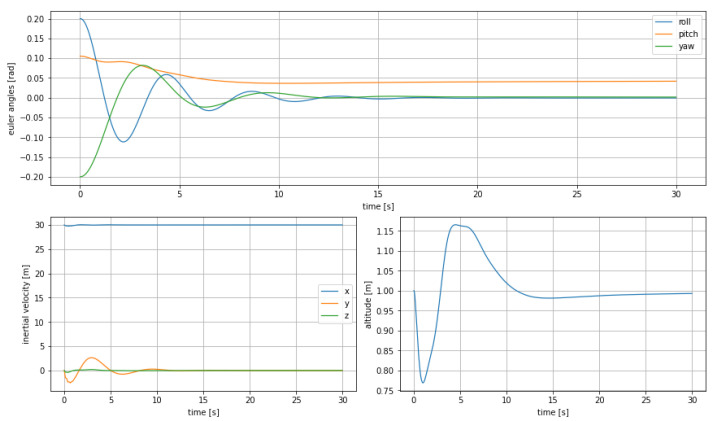
Euler angles (**top**), inertial velocities (**bottom left**) and altitude change (**bottom right**) in altitude change using 6 PID controllers.

The controllers are applied on the feedback linerised model shown in the previous section. After the feedback linearization process the part of the model that refers to the body referred dynamic is linear and decoupled, as proven by the zeros block in the upper-left part of the matrix (24). Therefore the system can be controlled in body frame by using one controller for each one of the body referred velocities, angular and linear, using the desired values as reference. Results are shown in Figure 14.

All the controllers use the controlled variable as reference, expect for the controller on the vertical body referred velocity $v_{b,z}$, which takes as reference the altitude *h*, so as to control it directly. Despite having been linearized, the system still has non-linearities in the relation between the altitude and the input used to control it, as explained more in detail later.

The Table 11 shows the evaluation parameters for this case.

Since a PID is meant to directly control a variable in a SISO (Single Input Single Output) system, in this case, due to the underlying nonlinearities and couplings aforementioned, its behaviour is rather different from a classic scenario. Controlling the altitude *h* through an input that acts on ${\dot{v}}_{b,z}$ without having a direct input on the altitude itself, means controlling a state by directly acting on its second derivative. For this reason the tuning of the controller PID parameters has been done empirically.

## 4. Discussion and Conclusions

### 4.1. Discussion on Controllers’ Performance

**Figure 15:**
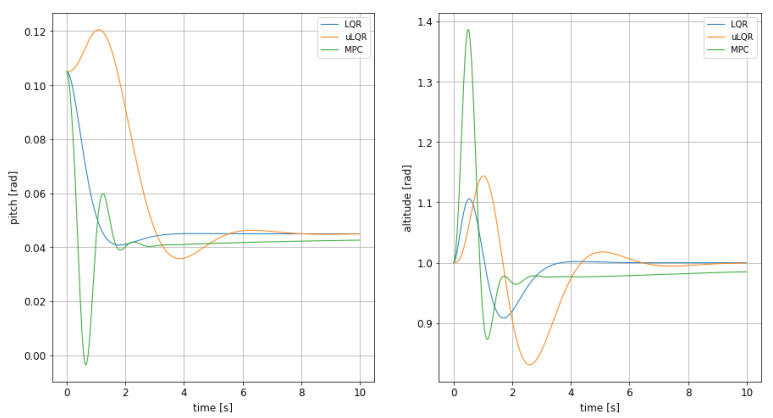
Comparison between the altitude and pitch dynamic for LQR, updating LQR and non-linear MPC.

The comparison between the four different control strategies, namely MPC, LQR, adaptive LQR and PID in a recovery from a disturbed attitude can be seen in Figure 15. The evolution of pitch and altitude show greatly different behaviours among the controllers, with the MPC and the simple LQR having a comparable one on altitude. The undershoot is of the same order of magnitude, around 10%, but the other two parameters used for comparison, settling time and overshoot, are quite distinct to each other.

The MPC shows the biggest overshoot amongst the three, over 38%. Even the adaptive LQR has a higher overshoot than the regular LQR, showing no improvement on haste. Most importantly the MPC is affected by the lack of perfect steady state tracking, a problem likely caused by the complicated expression of the input surfaces. If it were not for this it would be quite faster than the LQR, but instead its settling time is penalized strongly by this behaviour, almost reaching 7 s, much slower compared to the 2.87 s of the LQR.

The LQR is for sure the cleanest of them all, with a bounded oscillation and fast recovery time. Even the pitch behaviour, which is of secondary importance, is much smoother. It for sure wins for this scenario, where the more complex updating LQR does not seem to show any tangible benefit over the simple LQR, introducing more problems than it solves. The values supporting all these statements and conclusions are shown in Table 12, being all of them referred to the behaviour of the altitude.

The results obtained using the PID controller for recovery from a disturbed attitude show how this simple kind of controller is unsuitable for such a complex task. The undershoot is significantly larger than the other cases and the settling time is way too long to be considered as a usable control strategy. While comparing the different settling times it is important to recall that the MPC gets close to the steady state value despite maintaining an error during the transition.

Regarding the behaviour of the two tested controllers, MPC and adaptive LQR, in obstacle avoidance by hopping over at different heights, results have shown that both of them has different application scenarios. On one hand, MPC shows a better performance when the objective is a fast reaction to the obstacle presence, as it minimizes the rising and settling time at the altitude target. On the other hand, adaptive LQR has shown a better performance when flight comfort is an issue, as it generates a less aggressive maneuvering. In both cases, safety is guaranteed as undershoot values keeps a sufficient clearance at any time, preventing any contact of the WIG craft with the water surface, as can be seen in Figure 8 and Figure 12 and their corresponding tables.

### 4.2. Conclusions

WIGs have been studied for decades, but, up to now, control issues have remained largely unsolved, preventing this kind of vehicles to become a principal actor in autonomous vehicles based mobility solutions.

In this paper we have analyzed and compared three different control strategies for ground effect Unmanned Surface Vehicles.

Starting from a well known and broadly accepted model of this kind of vessels, we have developed different control solutions that address the challenging problem of implementing and tuning a set of robust automatic controllers that allow their safe operation.

We have tested different control strategies on two critical manoeuvres of a WIG-craft: obstacle avoidance by fly over and recovering heading after a disturbance. Non-linear MPC, regular and adaptive LQR control strategies have been tested in these cases.

The MPC has shown for sure a better overall behaviour, especially considering that the used inputs are more compliant with reality and that the reference model used for it is not simplified in any way. Therefore this results can be considered satisfactory and reliable despite being more oscillation prone. This problem has to be addressed by changing the formulation of the MPC and tuning. Even if it slowed down in terms of rise time it would be still quite acceptable and its performance is guaranteed.

The LQR instead relies on a simplified model that would need for other simplifications to be applied in order to evaluate the real performances of this strategy.

The performances of the adaptive version of the LQR do not show the expected improvements over the simple version and harder tasks with time varying parameters are not expected to be handled properly by it.

What has been said for the LQR also applies to the PID controller. Moreover the PID is particularly unsuitable for this scenario given that it is meant for SISO systems, whereas the case of study is a MIMO one. This makes the tuning process particularly hard.

Future work will be devoted to the development of scaled models for the purpose of performing wind tunnel experiments and progressing towards a fully operative scaled model, that will allow us to experimentally confirm the results and conclusions obtained through simulation and start actual flight control experiments.

The following abbreviations are used in this manuscript:

## Figures and Tables

**Table 1:** Representative values of height control for MPC.

	$t_{r}$	$t_{s,2\%}$	Overshoot
unbounded pitch	0.45s	0.94 s	7.5%

**Table 2:** Representative values of height control for modified MPC.

	$t_{r}$	$t_{s,2\%}$	Overshoot
unbounded pitch	0.45 s	0.94 s	7.5%
bounded pitch	1.16 s	2.22 s	4.9%

**Table 3:** Height overshoot for different altitudes for the MPC, rise.

	$t_{r}$	$t_{s,2\%}$	Overshoot
4 m	0.45 s	0.94 s	7.5%
3 m	0.45 s	0.94 s	7.0%
2 m	0.45 s	0.73 s	5.5%

**Table 4:** Height undershoot for different altitudes for the MPC, descent.

	$t_{r}$	$t_{s,2\%}$	Undershoot
4 m	0.43 s	1.39 s	35.0%
3 m	0.44 s	1.35 s	23.7%
2 m	0.44 s	1.03 s	12.0%

**Table 5:** Height overshoot for different altitudes for the MPC with constraints on control surfaces, rise.

	$t_{r}$	$t_{s,2\%}$	Overshoot
4 m	0.63 s	1.13 s	7.4%
3 m	0.53 s	0.87 s	7.5%
2 m	0.46 s	0.74 s	5.4%

**Table 6:** Height overshoot for different altitudes for the MPC with constraints on control surfaces, descent.

	$t_{r}$	$t_{s,2\%}$	Undershoot
4 m	1.12 s	1.72 s	15.5%
3 m	0.76 s	1.36 s	14.2%
2 m	0.46 s	1.08 s	12.0%

**Table 7:** Results of MPC recovering from a disturbed attitude.

	$t_{s,2\%}$	Undershoot	Overshoot
MPC	6.8 s	12.7 %	38.6%

**Table 8:** Results of height control with the adaptive version of LQR.

	$t_{r}$	$t_{s,2\%}$	Overshoot
Adaptive LQR	0.86 s	2.06 s	6.6%

**Table 9:** Height overshoot for different altitudes using adaptive LQR, rise.

	$t_{r}$	$t_{s,2\%}$	Overshoot
4 m	0.87 s	2.00 s	6.2%
3 m	0.87 s	1.96 s	5.6%
2 m	0.86 s	1.88 s	4.5%

**Table 10:** Height undershoot for different altitudes using adaptive LQR, descent.

	$t_{r}$	$t_{s,2\%}$	Undershoot
4 m	0.86 s	2.46 s	24.5%
3 m	0.86 s	2.36 s	16.7%
2 m	0.85 s	2.16 s	9.0%

**Table 11:** Comparison among all the tested controllers: recovering from a disturbed attitude.

	$t_{s,2\%}$	Undershoot	Overshoot
PID	9.98 s	23.2%	16.5%

**Table 12:** Comparison among all the tested controllers: recovering from a disturbed attitude.

	$t_{s,2\%}$	Undershoot	Overshoot
LQR	2.87 s	9.2%	10.6%
PID	9.98 s	23.2%	16.5%
Adaptive LQR	4.09 s	16.9%	14.4%
MPC	6.8 s	12.7%	38.6%
